# Burden of intestinal helminths and associated factors three years after initiation of mass drug administration in Arbaminch Zuria district, Southern Ethiopia

**DOI:** 10.1186/s12879-018-3330-3

**Published:** 2018-08-29

**Authors:** Getaneh Alemu, Zeleke Aschalew, Eshetu Zerihun

**Affiliations:** 1 Department of Medical Laboratory Science, College of Medicine and Health Sciences, Bahir Dar University, Bahir Dar, Ethiopia; 2 Department of Nursing, College of Medicine and Health Sciences, Arba Minch University, Arba Minch, Ethiopia; 3 Department of Public Health, College of Medicine and Health Sciences, Arba Minch University, Arba Minch, Ethiopia

**Keywords:** Prevalence, Helminth, School age children

## Abstract

**Background:**

Epidemiological information on the burden of various parasitic infections and associated factors in different localities is indispensable to develop appropriate control and prevention measures as well as to monitor and evaluate existing programs. Hence the aim of the present study was to assess the prevalence and associated factors of intestinal helminths among school age children in Arbaminch Zuria district, Southern Ethiopia.

**Methods:**

A community based cross-sectional study was conducted by recruiting 391 primary school age children. Participants were selected using simple random sampling technique. Pre-tested structured questionnaire was used to collect data about socio-demographic characteristics and perceived factors associated with intestinal parasitosis. Stool microscopic examination was done following standard protocol. Data were analyzed using Statistical Package for Social Science version 22.

**Results:**

Among 391 children participated, 181 (46.3%; 95% CI: 41.9–51.5) were infected with intestinal helminths. The magnitudes of single and double infections were 40.2% and 6.1% respectively. Seven helminths were detected among which *Ascaris lumbricoides* was with the highest frequency (56, 14.3%) followed by hookworms (55, 14.1%). Not washing fruits and vegetables before consumption (AOR = 2.16; 95%CI: 1.10–4.25, *p* = 0.026) and habit of swimming (AOR = 1.17; 95%CI: 1.03–1.95, *p* = 0.045) were significantly associated with helminthic infection.

**Conclusion:**

High prevalence of intestinal helminthic infections among school age children demands integration of deworming with water, hygiene and sanitation as well as consistent health education.

**Electronic supplementary material:**

The online version of this article (10.1186/s12879-018-3330-3) contains supplementary material, which is available to authorized users.

## Background

Globally about two billion people are infected with intestinal helminths mainly in resource-poor settings; children being the most affected population groups [1]. The big three intestinal helminths, commonly named soil transmitted helminths (STH), pose the most common parasitic infections worldwide. About 819 million people are infected with *Ascaris lumbricoides*, 465 million with *Trichuris trichiura*, and 439 million with hookworms [2].

Worldwide 800 million individuals are at risk of intestinal schistosomiasis; about 200 million people (160 million live in Sub-Saharan Africa) are estimated to be infected [3]. Ethiopia is one of the Sub-Sahara African countries with heavy burden of intestinal helminths. Soil transmitted helminth infections are distributed throughout the country that 81 million people live in endemic areas, of which, 25.3 million are school-age children (SAC). There are an estimated 38.3 million people (12.3 million are SAC) living in schistosomiasis endemic areas [4].

Intestinal helminths can infect all age groups but the magnitude and morbidity outweighs among primary SAC. Recent data show high burden of intestinal helminths among SAC (Table 1).

**Table 1:** Previous studies showing magnitude of intestinal parasites among school age children

Study Site (Citation)		Magnitude of Intestinal helminths	Laboratory Method used	Sample size
Nepal [5]		27.67%	Direct wet mount	495
Yemen [6]		90%	Direct wet mount and Formol-ether	330
Nigeria [7]		63.49%	Formol-ether	252
Sao Tome and Principe [8]		64.7%	Merthiolate-iodine-formaldehyde	252
Sudan [9]		24.9%	Kato-katz	662
Sudan [10]		84%	Direct wet mount and Formol-ether	120
Sudan [11]		30%	Formol-ether	200
Kenya [12]		44.05%	Kato-katz	377
Ethiopia	Bahir Dar [13]	65.5%	Formol-ether	359
	Zege [14]	69.1%	Formol-ether	408
	Maksegnit and Enfranz [15]	66.4%	Kato-katz	550
	Chencha [16]	36.8%	Kato-katz	408
	Arba Minch zuria [17]	39.9%	Direct wet mount and Formol-ether	858
	Hawassa [18]	67.9%	Formol-ether	374
	Wolyta [19]	72.2%	Kato-katz and Formol-ether	503
	Jimma Zone [20]	48.4%	Kato-katz	302

Helminth infections bring multiple health problems with a long term effect of growth retardation, reduced mental development, increased susceptibility to other infections and malnutrition, low academic performance and school absenteeism [5, 22]. Considering the health impact of both STH and schistosomiasis in the country, Ethiopia has launched a national deworming program in November 2015 giving priority to SAC. In the same year, 13 and 5 million SAC were treated for STH and schistosomiais respectively [4].

Arba Minch University, where all authors of the present study are affiliated, has been conducting independent monitoring of the national deworming program since 2015. One of the major challenges we observed was difficulty in accessing and convincing non-enrolled children to take drugs (unpublished data). Despite this, most of the previous deworming monitoring and coverage surveys were conducted in primary schools; excluding non-enrolled children. Moreover, the deworming program is not integrated with water, sanitation and hygiene (WASH) program as well as vector control (for Schistosomiasis) making re-infection common. Factors such as poor environmental sanitation and personal hygiene predispose people for intestinal parasitic infection. In addition, several occupational and socio-cultural factors affect the level of risk at different geographical settings [9, 19–21, 23, 24]. Except in one study [17], predisposing factors for intestinal parasitosis are also not well studied for primary school age children at community level. Hence the aim of the present study was to conduct a community based assessment on the burden of intestinal helminths and associated factors three years after initiation of the national mass drug administration (MDA) in Arbaminch Zuria district, Southern Ethiopia.

## Methods

### Study design and area

A community based cross-sectional study was conducted among SAC (5 to 14 years) living in Health Demographic Surveillance Site (HDSS) of Arbaminch Zuria district, Gamo Gofa Zone, Southern Ethiopia. The district lies surrounding Arbaminch town which is located about 454 km south of Addis Ababa, the capital of Ethiopia. The district is composed of a total of 31 kebeles (neighbourhood) with three different climatic zones: high land, midland and lowland. The HDSS include 9 kebeles representing all the three climatic zones.

### Sample size and sampling technique

**Fig. 1:**
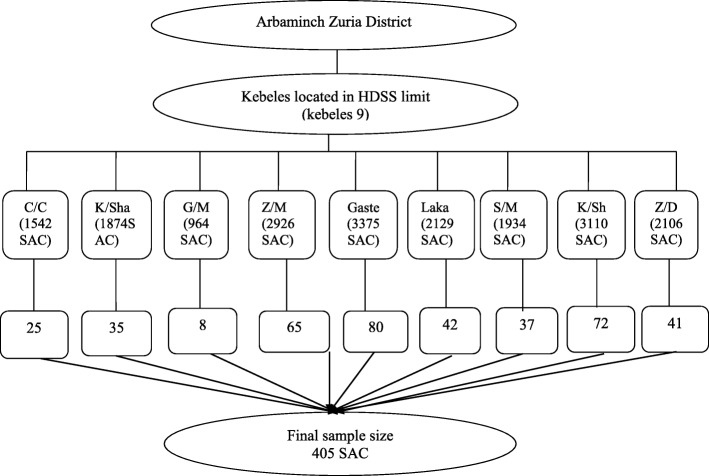
Schematic presentation of sampling procedure for the study on burden of intestinal helminths and associated factors among school age children in Arbaminch Zuria district, Southern Ethiopia, 2017. (C/C- Chano Chalba, K/Sha- Kola Shara, G/M- Genta Mecha, Z/M- Zigiti Merche, S/M- Shelle mella, K/Sh- Kolla shelle, Z/D- Zaise Dambille)

This study is part of a large scale project investigating ‘Undernutrition and Its Associated Factors among School Age Children in Arbaminch Zuria District’. Prevalence of undernutrition and perceived factors were considered to calculate the sample size of the project by referring previous findings [25–28]. Hence, 405 SAC were recruited to participate. All 9 kebeles found within the limit of HDSS were selected purposively to easily access a sampling frame of households with SAC. Proportional allocation based on the number of households with SAC was made (Fig. 1). Then, households with SAC were selected by simple random sampling technique. If there is only one eligible SAC in the selected household, he/she was involved in the study; however, if more than one eligible SAC are there, one was selected for the study by lottery method. Children who were critically ill during the time of data collection and those residing in the study area for less than 6 months were excluded from the study.

### Data collection

#### Socio-demographic characteristics

Medical laboratory technicians who are fluent speakers in the local language (Gamo) were trained for data collection. A pretested structured questionnaire was translated to local language and administered through face to face interview to caregivers (preferably mothers). It was used to collect data about socio- demographic characteristics and associated factors for intestinal helminthic infections (Additional file 1).

#### Stool examination

Each participating child was consulted about sample collection and supplied with labeled plastic stool cup, toilet paper and applicator stick and was instructed to bring about 5 g of stool. The collected sample was emulsified in 10% formal water and transported to the Microbiology and Parasitology laboratory of College of Medicine and Health Sciences, Arba Minch University for microscopic examination. About 1 g stool sample was concentrated and examined using the formol-ether concentration technique following standard protocol as expressed in our previous publication [29] .

### Statistical analysis

Data was edited, cleaned, entered and analyzed using SPSS version 22.0. Descriptive statistics were calculated to describe the study population characteristics. Bivariate logistic regression was used to assess associations between categorical variables. Multivariate regression model then followed for variables with *p* ≤ 0.25 in the bivariate analysis. Association between variables was considered statistically significant only if *p*-value ≤0.05 at 95% confidence level.

## Results

### Socio- demographic characteristics of study participants

Among 405 SAC recruited, 6 refuse to participate and 8 were unable to produce adequate amount of stool for parasitological examination. Hence data from 391 SAC were complete for analysis. One hundred ninety six (50.1%) were male and 195 (49.9%) were female children. The mean age of study participants was 10.1 years old with standard deviation of 2.6 (Table 2).

**Table 2:** Socio-demographic characteristics of school age children (*n* = 391) in Arbaminch Zuria district, Southern Ethiopia, 2017

Variables	Category	Frequency (%)
Age Group (In Years)	5–11	254 (65.0)
	12–14	137 (35.0)
Child Sex	Male	196 (50.1)
	Female	195 (49.9)
School Enrolment	Enrolled	223 (57)
	Non enrolled	168 (43)
Grade Level	1–4 class	169 (75.8)
	5–8 class	54 (24.2)
Birth Order	≤ 2	200 (51.2)
	> 2	191 (48.8)
Religion Of Parent/ Care Giver	Protestant	241 (61.6)
	Orthodox	142 (36.3)
	Others	8 (2.0)
Ethnicity Of Parent/ Care Giver	Gamo	338 (86.4)
	Zeise	35 (9.0)
	Wolaita	11 (2.8)
	Others	7 (1.8)
Educational Status Of mother	No formal education	257 (65.7)
	Unable to read and write	38 (9.7)
	Primary level	83 (21.2)
	Secondary level and above	13 (3.3)
Occupation Of Mother/ Care Giver	Housewife	370 (94.6)
	Government employee	1 (0.3)
	Private employee	14 (3.6)
	Others	6 (1.5)
Family Size	< 4	18 (4.6)
	≥4	373 (95.4)
Climatic Zone In Which The Household Is Located	Lowland	163 (41.7)
	Midland	44 (11.3)
	Highland	184 (47.1)
Illness in the last 2 weeks	Yes	16 (4.1)
	No	375 (95.9)
Habit of eating row fruits and vegetables	Yes	208 (53.2)
	No	183 (46.8)
wash fruits and vegetables before consumption	Yes	113 (54.3)
	No	95 (45.7)
Habit of swimming	Yes	108 (27.6)
	No	283 (72.4)
Habit of hand washing after contact with soil	Yes	229 (58.6)
	No	162 (41.4)
Habit of hand washing before meal	Yes	277 (70.8)
	No	114 (29.2)
Habit of hand washing after toilet	Yes	265 (67.8)
	No	126 (32.2)
Habit of wearing shoe	Yes	362 (92.5)
	No	29 (7.5)
Presence of domestic animals at home	Yes	338 (86.4)
	No	53 (13.6)
Dewormed in last year national deworming campaign	Yes	128 (32.7)
	No	263 (67.3)
Number of times taking deworming in the last year	Once	102 (79.7)
	Twice	26 (20.3)
Latrine availability	Yes	374 (95.7)
	No	17 (4.3)
Source of drinking water	Pipe water	303 (77.5)
	Protected well/ spring	36 (9.2)
	Unprotected well/ spring	37 (9.5)
	River	15 (3.8)

### Prevalence of intestinal Helminths

One hundred eighty two children (46.5%; 95% CI: 41.9–51.4) were infected with at least one intestinal parasite. *Giardia lamblia* cyst was the only intestinal protozoa detected; all other infections were caused by helminths. In this study, we will focus on prevalence of helminths (excluding protozoa) which was 46.3% (95% CI: 41.9–51.5). In total seven helminths were detected among which *A. lumbricoides* was with the highest frequency (56, 14.3%) followed by hookworms (55, 14.1%) and *Hymenolepis nana* (17, 4.3%) (Table 3).

**Table 3:** Prevalence of intestinal parasites among school age children (*n* = 391) in Arbaminch Zuria district, Southern Ethiopia, 2017

Parasites		Frequency	
		Number	Percentage
Helminths	*Ascaris lumbricoides*	56	14.3
	*Hookworms*	55	14.1
	*Hymenolepis nana*	17	4.3
	*Schistosoma mansoni*	12	3.1
	*Trichuris trichuria*	10	2.6
	*Taenia species*	4	1.0
	*Entrobius vermicularis*	3	0.8
Protozoa	*Giardia lamblia* cyst	1	0.3
Number of helminth Species per child	1	158	40.4
	≥2	24	6.1
Total Prevalence	Helminth	181	46.3
	Protozoa	1	0.2
	Helminth + Protozoa	182	46.5

### Factors associated with intestinal Helminth infection

The bivariate analysis show that prevalence of intestinal helminths was higher in children with age group of 6–11 (48.8%) years old as compared to 12–14 (42.3%) years old children; however the difference was not statistically significant (*p* = 0.221). Latrine availability was strongly associated with intestinal parasitosis according to the bivariate analysis result (COR = 1.25; 95%CI: 1.08–1.79, *p* = 0.018) but that was not the case when corrected for confounders in the multivariate analysis (AOR = 1.43; 95%CI: 0.10–1.95, *p* = 0.275). In the multivariate regression model, not washing fruits and vegetables before consumption (AOR = 2.16; 95%CI: 1.10–4.25, *p* = 0.026) and habit of swimming (AOR = 1.17; 95%CI: 1.03–1.95, *p* = 0.045) were significantly associated with intestinal parasitic infection (Table 4).

**Table 4:** Factors associated with intestinal parasitic infection among school age children (*n* = 391) in Arbaminch Zuria district, Southern Ethiopia, 2017

Variables	Category	Number examined	Rate of helminth infection N (%)	COR (95%CI)	*p*-value	AOR (95%CI)	*p*-value
Age group (in years)	6–11	254	124(48.8)	0.77 (0.51–1.17)	0.221	0.59 (0.32–1.11)	0.103
	12–14	137	58 (42.3)	1		1	
Child sex	Male	196	90 (45.9)	1			
	Female	195	92 (47.2)	0.95 (0.64–1.41)	0.803		
School Enrolment	Enrolled	223	109(48.9)	1			
	Non enrolled	168	73 (43.5)	1.24 (0.83–1.86)	0.287		
Wash fruits and vegetables before consumption	Yes	113	63 (55.8)	1	1		
	No	95	44 (46.3)	1.46 (0.84–2.53	0.176	2.16 (1.10–4.25)	0.026
Habit of swimming	Yes	108	61 (56.5)	1.15 (1.03–1.69)	0.015	1.17 (1.03–1.95)	0.045
	No	283	121 (42.8)	1		1	
Habit of hand washing after toilet	Yes	265	118 (44.5)	1		1	
	No	126	64 (50.8)	0.78 (0.51–1.19)	0.246	0.73 (0.35–1.54)	0.408
Hand washing habit before meal	Yes	308	130 (46.9)	1		1	
	No	114	52 (45.6)	1.05 (0.68–1.63)	0.812	0.74 (0.31–1.75)	0.493
Habit of wearing shoe	Yes	362	167 (46.1)	1			
	No	29	15 (51.7)	0.97 (0.44–2.13)	0.933		
Presence of tame animals at home	Yes	338	161 (47.6)	0.72 (0.40–1.30)	0.278		
	No	53	21 (39.6)	1			
Dewormed in last year national deworming campaign	Yes	128	68 (53.1)	1		1	
	No	263	114 (43.3)	1.48 (0.97–2.26)	0.070	1.23 (0.68–2.22)	0.496
Latrine availability	Yes	374	169 (45.2)	1		1	
	No	17	13 (76.5)	1.25 (1.08–1.79)	0.018	1.43 (0.10–1.95)	0.275
Source of drinking water	Safe	339	158 (46.6)	1		1	
	Unsafe	52	24 (46.2)	1.02 (0.57–1.83)	0.951	1.42 (0.54–3.73)	0.481

*AOR* Adjusted Odd Ratio, *CI* Confidence Interval, *COR* Crude Odd Ratio

## Discussion

Prevalence of intestinal helminth infection in the present study was comparable with 44.05% prevalence in Kenya, 44.2% in Tilili and 48.4% in Jima zone [12, 20, 23]. Lower prevalences ranging from 24.7 to 39.9% were reported from three separate studies (Tepi, Bale, Chencha,) in Ethiopia [16, 21, 24]. Inclusion of non-enrolled children in the present study might increase the rate of infection because most of non-enrolled children were not dewormed in the previous three rounds of national deworming (unpublished data). Intestinal helminths are more prevalent in areas with hot and humid climate accompanied with poor sanitation. Both Chencha and Bale are highlands with very cold weather condition which delays the transmission of intestinal helminths.

The same study conducted in 2010/11 by Wogayehu et al. shows a prevalence of 39.9% in Arba Minch Zuria district [17]. After 3 years of bi-annual deworming, we reported increased prevalence in the area (46.5%). Re-infection due to ineffective implementation of WASH and low deworming coverage for non-enrolled children could justify for the increased prevalence of intestinal helminths. Variations in data collection time (season) and sample size also influence magnitude of infection. Wogayehu et al. collected the data for 11 consecutive months (September to July) in order to avoid seasonal variations and the sample size was 858, which is more representative as compared to the present study [17].

Gawad et al. reported an intestinal parasite infection rate of 90% in Yemen [6]. Higher prevalences of 64.4% and 84% were also reported in two separate studies by Abdulshafi et al.*..* and Siddig et al. respectively from Sudan [10, 11]. In all the three studies, stool samples were processed for direct wet mount to detect intestinal protozoa; we couldn’t do this because we collected data house to house which makes difficult to prepare and examine direct wet mount smear immediately after stool collection.

Gawad et al. performed duplicate Kato Katz technique which increases the sensitivity of detecting helminth ova specially Schistosoma. Findings by Nasiru et al also reveal higher prevalence of intestinal parasitosis (63.49%) from Nigeria [7]. Inter-country variations in distribution of intestinal parasites and sample size plus method of detection might bring such differences (Table 1).

Higher prevalences of 65.5% to 72.2% were reported in separate studies from Bahir Dar, Maksegnit and Enfranz, Hawassa, Zege and Wolyta [13–15, 18, 19]. Variation in spatial distribution of intestinal helminths, number of study participants recruited and the laboratory techniques applied brought this difference. Moreover, data for the previous studies conducted in Ethiopia were collected before or early after the national deworming program (2015) was implemented in the country [13, 14, 18, 19].

*Ascaris lumbricoides* was the most frequent intestinal helminth detected. This is in line with the global data denoting that *A. lumbricoides* is the most prevalent helminth in the world with 819 million cases annually [2]. The same is true in the Ethiopian context as supported by previous studies from different parts of the country [16–18, 20, 23]. *S. mansoni* causes the most common helminthic infection according to studies from Gondar and Wolayta, Ethiopia likely due to variation in geographical distribution of *S. mansoni* [15, 19].

Among all study participants, 24 (6.1%) acquired two parasites or more. This is much lower as compared to previous findings from Ethiopia. Results from Bahir Dar, Zegie and Hawassa reveal 16.2%, 25% and 25.7% of the study participants were infected at least with two intestinal parasites respectively [13, 14, 18]. The fact that we didn’t detect intestinal protozoa by direct wet mount examination of fresh stool lowers the co-infection rate in the present study. The difference in prevalence of multiple parasitic infections at a time might be varied in relation to level of environmental contamination, level of awareness about parasitic infection and socioeconomic factors [13].

The present study showed that not washing fruits and vegetables before consumption and habit of swimming were strongly associated with the presence of intestinal parasitic infections. Intestinal helminths, whose ova are infective stages, are acquired by ingestion with contaminated food and water as well as oral contact with hands. Except for hookworm and schistosomiasis which penetrates the skin directly, fruits and vegetables eaten in raw are common sources of infection. Hence, SAC who eat fruits and vegetables without washing were 2 times at higher risk of being infected with intestinal parasites as compared to those who washed fruits and vegetables before consumption. Parasitic contamination rates ranging from 22.22 to 57.8% of fruits and vegetables sold at local markets and at pre-harvest phase have been reported in Ethiopia [30–32]. The present study is in agreement with previous results in Ethiopia [29].

The infective cercaria of schistosoma parasites develop in fresh water dwelling snails. Hence frequent contact with contaminated water poses risk of acquiring schistosomiasis. SAC who frequently swim in rivers and lakes were 1.17 times at higher risk of being infected with intestinal parasites compared to SAC having no habit of swimming. Begna et al. also reported similar finding from Eastern Ethopia that water contact activities predispose for intestinal parasitosis [24]. Hook worms easily infect people who walk barefoot. Hence previous studies show that shoe wearing habit is associated with hook worm infection [13, 23, 24]; but in the present study, the association was not significant. Frequent contact with soil despite shoe wearing and other confounding factors might alter the trend. In previous studies water source, habit of hand washing after toilet and before meal, gender, availability and utilization of latrine were found to be associated with intestinal parasitosis [13, 15, 18, 20, 23, 24]. However, all these factors were not associated with intestinal parasitosis in the present study. Hence, large scale study recruiting more number of participants is required in order to drive definitive conclusion.

## Conclusion

Despite 3 years of bi-annual deworming program implementation, intestinal hellminths are still major public health problems in Arba Minch Zuria district. Hence we strongly recommend integration of deworming with WASH and vector control programs in order to control intestinal helminthiasis in Ethiopia and honestly, deworming everyone.

## Additional file

Additional file 1: Questionnaire administered to assess the Burden of intestinal helminths and associated factors 3 years after initiation of Mass drug administration in Arbaminch Zuria district, Southern Ethiopia, 2017. The data contains list of questions asked to children (study participants) in order to collect socio-demographic data and factors associated with intestinal helminth infection. (DOCX 20 kb)

## Abbreviations

HDSS: Health Demographic Surveillance Site
SAC: School-Age Children
STH: Soil Transmitted Helminths
WASH: Water Hygiene and Sanitation
