# Supplementary material for: Burden of intestinal helminths and associated factors three years after initiation of mass drug administration in Arbaminch Zuria district, Southern Ethiopia
Source: BMC Infect Dis. 2018 Aug 29;18:435. doi: 10.1186/s12879-018-3330-3 (PMC6114701; doi:10.1186/s12879-018-3330-3)
Supplement: Supplementary file 1 — Questionnaire administered to assess the Burden of intestinal helminths and associated factors 3 years after initiation of Mass drug administration in Arbaminch Zuria district, Southern Ethiopia, 2017. The data contains list of questions asked to children (study participants) in order to collect socio-demographic data and factors associated with intestinal helminth infection. (DOCX 20 kb) [file 12879_2018_3330_MOESM1_ESM.docx]

Questionnaire administered to assess the Burden of intestinal helminths and associated factors three years after initiation of Mass drug administration in Arbaminch Zuria district, Southern Ethiopia, 2017.

Participant code ____________ Name of the kebele ____________Date__________

| **No.** | **Question** | **Answer** | **Code** | **Skip to** |
| --- | --- | --- | --- | --- |
| 001 | Age of the child |  | _ |  |
| 002 | Sex of the child | Male  Female | 1  2 |  |
| 003 | Educational level of care giver (mother) | Illiterate  Primary  Secondary /above | 1  2  3 |  |
| 004 | How many family members are there in your house? |  | | |
| 005 | Where do you get your food? | Cooked at home  From hotel  Some times at home some times in hotel  Other | 1  2  3  4 |  |
| 006 | Do you have habit of eating raw fruits/vegetables? | Yes, frequently  Yes, sometimes  No | 1  2  3 | →008 |
| 007 | Do you wash fruits and vegetables before eating in raw? | Yes, always  Yes, occasionally  No | 1  2  3 |  |
| 008 | Do you have habit of eating raw meat? | Yes, always  Yes, occasionally  No | 1  2  3 |  |
| 009 | Do you have habit of eating raw fish? | Yes, frequently  Yes, occasionally  No | 1  2  3 |  |
| 010 | Do you have hand washing habit after soil contact? | Yes with water and soap  Yes only with water  No | 1  2  3 |  |
| 011 | Do you have hand washing habit in-between handling raw & cooked food? | Yes with water and soap  Yes only with water  No | 1  2  3 |  |
| 012 | Do you have hand washing habit before eating? | Yes with water and soap  Yes only with water  No | 1  2  3 |  |
| 013 | Do you have hand washing habit after toilet? | Yes with water and soap  Yes only with water  No | 1  2  3 |  |
| 014 | Do you have hand washing habit after touching toilet materials? | Yes with water and soap  Yes only with water  No | 1  2  3 |  |
| 015 | Where do you get water for washing your clothes and house utensils? | Pipe water  River  Lake  other | 1  2  3  4 |  |
| 016 | Where do you get water for bathing? | Pipe water  River  lake  other | 1  2  3  4 |  |
| 017 | Where do you get water for drinking? | Pipe water  River  Lake  other | 1  2  3  4 |  |
| 018 | Do you have swimming habit? | Yes, frequently  Yes, occasionally  No | 1  2  3 | →020 |
| 019 | Where do you swim? | River  Lake  Swimming pool  other | 1  2  3  4 |  |
| 020 | Do you have shoe wearing habit? | Yes, always  Yes, occasionally  No | 1  2  3 |  |
| 021 | Do you (your family) raise cattle? | Yes  No | 1  2 |  |
| 022 | Do you raise (your family) sheep/goat? | Yes  No | 1  2 |  |
| 023 | Do you have cat/dog in your compound? | Yes  No | 1  2 |  |
| 024 | Do you have separate house for cattle/sheep/goat? | Yes  No | 1  2 |  |
| 025 | Do you have latrine? | Yes  No | 1  2 | →027 |
| 026 | Ownership of the latrine | Private for households  Shared with neighbours | 1  2 |  |
| 027 | Do you have habit of using night soil for farming? | Yes  No | 1  2 |  |
| 028 | Do you have separate bath room? | Yes  No | 1  2 |  |
| 029 | How do you use toilet materials (towel, soap...)? | Privately  Common for the family | 1  2 |  |
| 030 | Did you have history of diarrhoea in the last 3 months? | Yes  No | 1  2 | →033 |
| 031 | Time of last diarrhoea occurrence | Before a month  Before 2 weeks  Within 2 weeks | 1  2  3 |  |
| 032 | Duration of last diarrhoea occurrence | More than a month  < a month  < a week | 1  2  3 |  |
| 033 | Did you have other gastro-intestinal discomfort in the last 3 months? | Yes  No | 1  2 |  |
| 034 | Have you visited a health facility during your last GIT problem? | Yes  No | 1  2 | →end |
| 035 | Were you told that you have got intestinal parasitosis during your visit? | Yes  No | 1  2 |  |
| 036 | Have you taken anti–parasitic drug? | Yes  No | 1  2 |  |
